# The effect of hypercapnia on static cerebral autoregulation

**DOI:** 10.14814/phy2.12059

**Published:** 2014-06-27

**Authors:** Blake G. Perry, Samuel J. E. Lucas, Kate N. Thomas, Darryl J. Cochrane, Toby Mündel

**Affiliations:** 1 School of Sport and Exercise, Massey University, Palmerston North, New Zealand; 2 School of Sport, Exercise and Rehabilitation Sciences, College of Life and Environmental Sciences, University of Birmingham, Birmingham, UK; 3 Department of Physiology, University of Otago, Dunedin, New Zealand; 4 School of Physical Education, Sport and Exercise Sciences, University of Otago, Dunedin, New Zealand; 5 Department of Surgical Sciences, University of Otago, Dunedin, New Zealand

**Keywords:** Cerebral blood flow, hypercapnia, lower body positive pressure, static cerebral autoregulation

## Abstract

Hypercapnia impairs cerebrovascular control during rapid changes in blood pressure (BP); however, data concerning the effect of hypercapnia on steady state, nonpharmacological increases in BP is scarce. We recruited fifteen healthy volunteers (mean ± SD: age, 28 ± 6 years; body mass, 77 ± 12 kg) to assess the effect of hypercapnia on cerebrovascular control during steady‐state elevations in mean arterial BP (MAP), induced via lower body positive pressure (LBPP). Following 20 min of supine rest, participants completed 5 min of eucapnic 20 and 40 mm Hg LBPP (order randomized) followed by 5 min of hypercapnia (5% CO_2_ in air) with and without LBPP (order randomized), and each stage was separated by ≥5 min to allow for recovery. Middle cerebral artery blood velocity (MCAv), BP, partial pressure of end‐tidal carbon dioxide (P_ET_CO_2_) and heart rate were recorded and presented as the change from the preceding baseline. No difference in MCAv was apparent between eupcapnic baseline and LBPPs (grouped mean 65 ± 11 cm·s^−1^, all *P *>**0.05), despite the increased MAP with LBPP (Δ6 ± 5 and Δ8 ± 3 mm Hg for 20 and 40 mm Hg, respectively, both *P *<**0.001 vs. baseline). Conversely, MCAv during the hypercapnic +40 mm Hg stage (Δ31 ± 13 cm·s^−1^) was greater than hypercapnia alone (Δ25 ± 11 cm·s^−1^, *P *=**0.026), due to an increased MAP (Δ14 ± 7 mm Hg, *P *<**0.001 vs. hypercapnia alone and *P *=**0.026 vs. hypercapnia +20 mm Hg). As cardiac output and P_ET_CO_2_ were similar across all hypercapnic stages (all *P *>**0.05), our findings indicate that hypercapnia impairs static autoregulation, such that higher blood pressures are translated into the cerebral circulation.

## Abstract (precis)

This experiment investigated the effects of Hypercapnia on static cerebral autoregulation in healthy humans using lower body positive pressure mediated increases in MAP. It was found that nonpharmacological increases in MAP are translated to the cerebral circulation during hypercapnia.

## Introduction

The control of the cerebral circulation is complex and is modulated by many factors, the most potent of which is the partial pressure of arterial carbon dioxide (PaCO_2_; Ogoh and Ainslie 2009). Alterations in PaCO_2_ result in pronounced cerebrovascular responses, with increased PaCO_2_ (hypercapnia) dilating cerebral resistance vessels leading to an increase in cerebral blood flow (CBF) and reductions in PaCO_2_ (hypocapnia) constricting vessels and reducing CBF (Kety and Schmidt 1948). This mechanism acts to maintain central pH with the resultant changes in CBF altering CO_2_, and thus [H^+^], washout from the brain (Ainslie and Duffin 2009). Another key modulator is mean arterial pressure (MAP), which in part determines cerebral perfusion pressure (CPP = MAP− intracranial pressure). Whilst the cerebral vasculature does possess an intrinsic ability to defend against changes in blood pressure (Lassen 1959), both steady‐state (static; Zhang et al. 2000; Lucas et al. 2010; Liu et al. 2013) and transient (dynamic; Tiecks et al. 1995b; Edwards et al. 2002; Claassen et al. 2009) changes in CPP result in concomitant perturbations in CBF.

The regulation of the cerebral circulation is further complicated by the interaction between MAP and PaCO_2_. Hypercapnia increases sympathetic discharge and elevates blood pressure via the chemoreflex (Morgan et al. 1995). Whilst the majority of the observed increase in CBF is due to the direct vaso‐active effect of CO_2_, the hypercapnic‐induced vasodilation reduces the efficacy of dynamic cerebral regulation and subsequently the ability to defend against dynamic changes in CPP (Aaslid et al. 1989; Maggio et al. 2013). Thus, the chemoreceptor‐mediated elevation in CPP increases middle cerebral artery blood flow velocity (MCAv) over and above that induced by the hypercapnic‐induced vasodilation alone (Przybylowski et al. 2003; Ainslie et al. 2005; Claassen et al. 2007) via a pressure‐passive effect (Battisti‐Charbonney et al. 2011). Similar pressure‐passive results have been shown during impaired static cerebral autoregulation when pharmacological manipulations in MAP have been induced (Tiecks et al. 1995a), although data using nonpharmacological interventions in healthy humans is limited.

Recently, we have shown that steady‐state increases in MAP can be induced via lower body positive pressure (LBPP) and appear to challenge ‘static’ cerebral autoregulation (Perry et al. 2013). That is, moderate elevations in MAP were associated with concomitant increases in CBF. Importantly, this methodology avoids possible pharmacologically induced changes in MCA diameter (Ogoh et al. 2011). The purpose of this study was to investigate the effect of hypercapnia on cerebrovascular regulation during steady‐state increases in MAP (static cerebral autoregulation) induced by LBPP. The hypothesis for this experiment was that hypercapnia would impair static cerebral autoregulation, such that increases in MCAv would occur concomitantly with MAP during LBPP.

## Methods

### Participants

Fifteen healthy participants were recruited for this study (11 males, four females, mean ± SD: age, 28 ± 6 years; body mass, 77 ± 12 kg; height, 175 ± 7 cm). Each participant was fully informed of all potential risks and experimental procedures, after which written consent was obtained. All experimental procedures and protocols were approved by the University's Human Ethics Committee and performed in accordance with the *Declaration of Helsinki*. All participants were free from cardiovascular and cerebrovascular disease, were nonsmokers and were not taking medication (aside from oral contraceptive). Participants arrived at the laboratory for the familiarization and experimental trials hydrated (urine specific gravity 1.008 ± 0.006) and having abstained from strenuous exercise, alcohol and caffeine for at least 24 h.

### Measurements

Blood flow velocity in the right middle cerebral artery (MCAv) was measured using a 2‐MHz pulsed Doppler ultrasound system (DWL; Compumedics Ltd, Singen, Germany), secured with a plastic headband device (DWL) to maintain a constant insonation angle. The partial pressure of end‐tidal carbon dioxide (P_ET_CO_2_) was sampled at the mouth using a gas analyser (ML206; ADInstruments, Bella Vista, NSW, Australia). Blood pressure was measured noninvasively using finger photoplethysmography (Finapres Medical Systems; Biomedical Instruments, Amsterdam, The Netherlands) and heart rate via three lead electrocardiogram (ADInstruments). Finger blood pressure values were checked against an automated sphygmomanometer initially and regularly during baseline periods. If the two were not in agreement the finger cuff was replaced and/or the hand was warmed until the pressures matched. All data were acquired continuously via an analog‐to‐digital converter (PowerLab ML870; ADInstruments) at 1000 Hz. Data were displayed in real time using commercially available LabChart software (v7.3.3; ADInstruments) and recorded for subsequent off‐line analysis.

In a subset of five participants, the internal carotid artery (ICA) diameter was measured during the last minute of the baseline and each steady‐state stage. A B‐mode image of the ICA was obtained in longitudinal section and the diameter was measured approximately 2 cm distal to the carotid bifurcation. All ultrasound examinations were performed by the same Vascular Technologist (K.N.T.) on an ultrasound machine (Terason 3000; Teratech, Burlington, MA) with a 10 MHz linear array transducer. Ultrasound settings (depth, focus position, gain and compression) were optimized for each participant and these were kept consistent throughout each examination. Care was taken to ensure the transducer was stable. At least 15 cardiac cycles were used to obtain average data for diameter. Cine loops were recorded as AVI files for offline analysis using an edge‐detection software program, Cardiovascular Suite UE v 2.5 (Quipu, Pisa, Italy).

Cardiac output (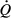) was calculated from the blood pressure waveform using the Modelflow method (Wesseling et al. 1993; BeatScope 1.02 software; Biomedical Instruments). Mean blood flow velocity (MCAv_mean_) and MAP were calculated as the integral for each cardiac cycle divided by the corresponding pulse interval. Systolic flow velocity was taken as the peak during one cardiac cycle and diastolic flow the lowest. An index of total peripheral resistance (TPRi) was estimated using the equation MAP/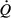, and cerebral vascular conductance (CVC) via the equation MCAv_mean_/MAP. The cerebrovascular reactivity to CO_2_ was calculated as the absolute ΔMCAv/ΔP_ET_CO_2_.

### Experimental protocol

**Figure 1.**
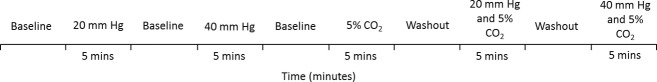
Experimental protocol. All experiments were conducted in the above order, however, the lower body positive pressure stages were randomized. Baseline and washout periods lasted until all variables had returned to initial baseline levels.

Participants visited the laboratory on two occasions, one familiarization and one experimental trial. Experimental trials were conducted in the supine position in a LBPP chamber at an ambient temperature of 19–22°C, relative humidity of 40–50% and barometric pressure of 758 ± 8 mm Hg. The experimental protocol is outlined in Figure 1. Following instrumentation and 20 min of supine rest, baseline values of all measures were recorded. All LBPP and hypercapnia (5% CO_2_ in air) stages were 5 min in duration with the order of the LBPP stages randomized. Baseline and washout periods lasted until all variables had returned to initial baseline levels (≥ 5 min).

Participants lay supine in a custom‐made LBPP box, sealed distal to the iliac crest. Pressure was produced via two commercially available vacuum cleaners, measured (in mm Hg) via a calibrated pressure transducer mounted within the box and controlled via a manual bleed valve. By design, participants’ P_ET_CO_2_ was matched between each respective stage; that is, P_ET_CO_2_ was matched within eucapnic and hypercapnic stages. Hypercapnia was induced using 5% CO_2_ in air that was breathed from a Douglas bag. Eucapnia was achieved by a researcher giving the subject verbal breathing instructions during testing to maintain P_ET_CO_2_ at baseline values.

### Data analyses

Baseline data were acquired in the last minute of the baseline period preceding each stage, and presented as the mean across that minute. Similarly, data were averaged in the last minute of each stage (hypercapnia alone, LBPP [20 and 40 mm Hg LBPP] and hypercapnia + LBPP [5% CO_2_ + 20 mm Hg LBPP and 5% CO_2_ + 40 mm Hg LBPP]). Inferential statistical analyses of dependent variables were analyzed using a two‐way analyses of variance (ANOVA; pressure [0, 20, 40 mm Hg LBPP] × CO_2_ [eupcapnia, 5% CO_2_]) for change from the preceding baseline period. The cerebrovascular reactivity to CO_2_ was determined between hypercapnic stages using a one‐way ANOVA. Data were assessed for approximation to a normal distribution and sphericity, with no corrections required. When a significant *F*‐value was observed (a priori set at *P *≤**0.05), *post‐hoc* pairwise comparisons (Bonferroni corrected) were performed. All data were analyzed using SPSS statistical software (v20; Chicago, IL) and presented as the mean ± SD, unless otherwise denoted.

## Results

**Figure 2.**
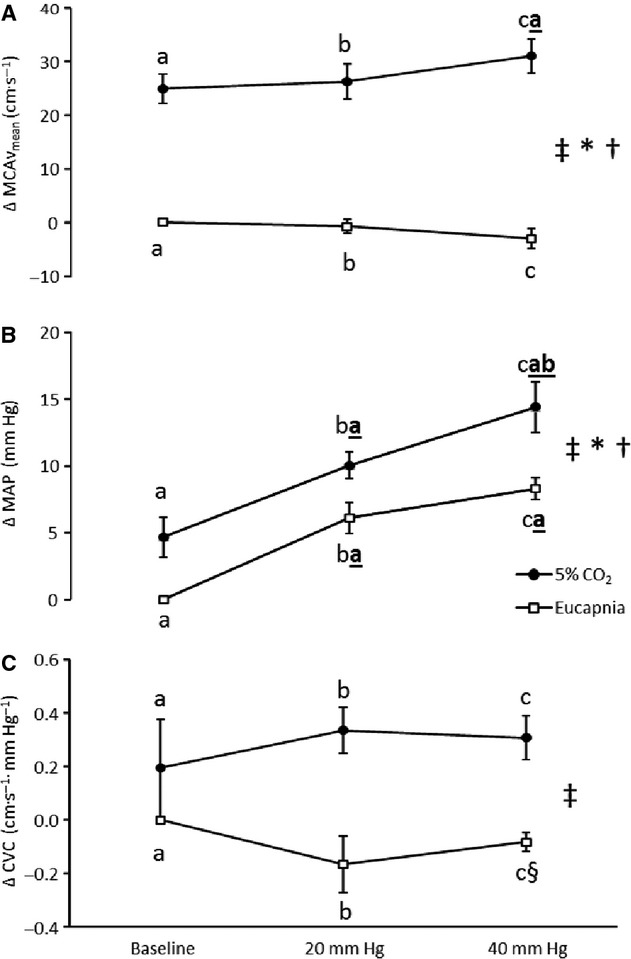
Absolute changes from baseline for mean middle cerebral artery blood flow velocity (MCAv_mean_, A), mean arterial blood pressure (MAP, B) and cerebrovascular conductance (CVC, C). The 0 reference on the y‐axis represents the eucapnic baseline values for each variable. The letters a, b, and c represent the lower body positive pressure levels baseline (no pressure), 20 and 40 mm Hg, respectively. Bolded and underlined letters represent differences between these pressure stages within each CO_2_ trial (*P *<**0.05). ^‡^Significant main effect of pressure, *P *≤**0.05; *significant main effect of CO_2_, *P *≤**0.05; ^†^pressure‐by‐CO_2_ interaction, *P *≤**0.05, ^§^trend to be different from baseline *P *=**0.06. Values are means ± SE.

**Figure 3.**
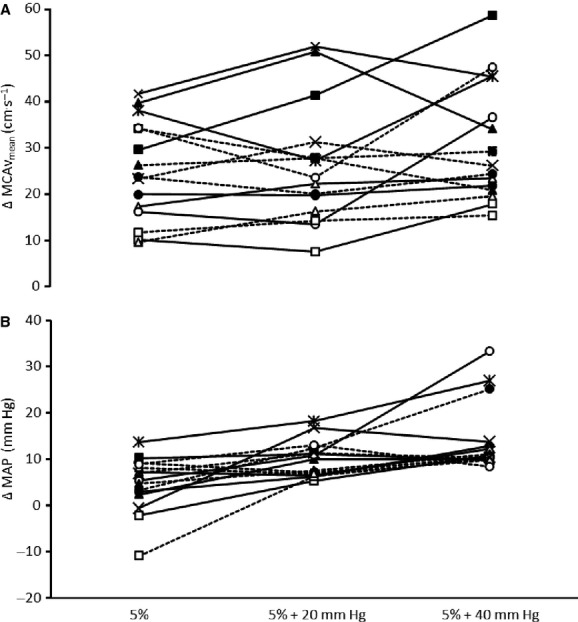
Individual mean middle cerebral artery blood flow velocity (MCAv_mean_, A) and mean arterial blood pressure (MAP, B) responses to 5% CO_2_ (5%) alone and in combination with 20 and 40 mm Hg of lower body positive pressure. The 0 reference on the y‐axis represents the eucapnic baseline values. Individuals are represented by the same symbol in both graphs.

Absolute changes from eucapnic baseline for MCAv_mean_, MAP and CVC are displayed in Figure 2. In summary, we observed a differential effect of LBPP‐induced increases in MAP on MCAv_mean_ with and without hypercapnia (interaction: *P* < 0.001). Specifically, MCAv was not altered from baseline during both LBPP stages in eucapnic conditions, despite the increased MAP with LBPP (Δ6 ± 5 and Δ8 ± 3 mm Hg for 20 and 40 mm Hg LBPP, respectively, both *P *<**0.001 vs. baseline; Fig. 2). In contrast, the hypercapnic‐induced increases in MCAv_mean_ (*P *<**0.001) were greater during the 40 mm Hg LBPP stage (Δ31 ± 13 cm·s^1^ mm Hg^−1^) compared to hypercapnia alone (Δ25 ± 11 cm·s^1^ mm Hg^−1^; *P *=**0.026, Fig. 2A), which was consistent with the greater elevation in MAP during the 40 mm Hg stage (Δ14 ± 7 mm Hg) compared to 20 mm Hg LBPP (Δ10 ± 4 mm Hg, *P *=**0.026) and hypercapnia alone (Δ5 ± 6 mm Hg, *P *<**0.001). Consequently, CO_2_ reactivity was greatest for the 40 mm Hg LBPP stage (3.8 ± 1.3 cm·s^1^ mm Hg^−1^/mm Hg) compared to hypercapnia alone (3.0 ± 1.0 cm·s^−1^/mm Hg; *P *=**0.029) and the 20 mm Hg LBPP stage (3.2 ±1.0 cm·s^1^ mm Hg^−1^/mm Hg; *P *=**0.070). Furthermore, CVC was unchanged between hypercapnic stages (*P *=**0.65; Fig. 2C). Individual responses are displayed in Figure 3.

Finally, the diameter of the ICA demonstrated small changes during each condition. Changes from baseline for eucapnic 20 and 40 mm Hg LBPP were −0.9 ± 5.7% and +4.9 ± 4.5%, respectively. ICA diameter increased +3.9 ± 3.5% for hypercapnia alone and +3.4 ± 4.0% and +3.2 ± 3.6% for hypercapnia 20 mm Hg and hypercapnia 40 mm Hg LBPP, respectively.

## Discussion

We investigated the effects of hypercapnia on MCAv during steady‐state, nonpharmacological increases in MAP. We used LBPP as a means of inducing modest increases in MAP. The main findings were that hypercapnia impaired cerebrovascular control of blood flow (velocity) during steady‐state increases in MAP such that MCAv increased concomitantly with blood pressure at greater LBPPs. The increase in MCAv_mean_ was mediated via elevations in both systolic and diastolic flow velocities and despite the elevated MAP, CVC remained unchanged. Thus, consistent with our hypothesis, hypercapnia impaired steady‐state CBF regulation.

Recently the efficacy of both static (Lucas et al. 2010; Perry et al. 2013) and dynamic autoregulation (Edwards et al. 2002; Claassen et al. 2009) has been scrutinized, with evidence for a cerebral circulation being much more pressure passive than originally described (Lassen 1959). Furthermore, there is evidence to indicate that hypercapnia impairs dynamic cerebral autoregulation (Aaslid et al. 1989; Zhang et al. 1998; Ainslie et al. 2005; Maggio et al. 2013). Przybylowski et al. (2003) ablated the chemoreceptor‐induced MAP response to apnoea via ganglionic blockade and demonstrated that the increase in MCAv is partially attributed to the increase in CPP rather than CO_2_ *per se*. Thus, when the hypercapnic stimulus is sufficient to induce elevations in MAP, increases in MCAv occur concurrently (Battisti‐Charbonney et al. 2011). Therefore, these results are complementary to those observed during dynamic testing. As cardiac output (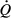), another modulator of MCAv (Ogoh et al. 2005), and P_ET_CO_2_ were unchanged across the hypercapnic stages (Table 1), it appears that the nonpharmacologically induced hypertension elevated MCAv_mean_ independently of CO_2_ and 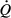.

**Table 1.** Changes from baseline during hypercapnia and lower body positive pressure.

Variable	Condition	Baseline	Δ From baseline		*P* values		
			20 mm Hg	40 mm Hg	Pressure	CO_2_	Pressure × CO_2_
Systolic MCAv, cm·s^−1^	Eucapnia	98 ± 17^a^	0 ± 5 (0 ± 5)^b^	−3 ± 8 (−3 ± 7)^c^	0.48	<0.001	0.014
	5%	28 ± 13 (28 ± 13)^a^	31 ± 14 (33 ± 14)^b^	36 ± 17 (39 ± 18)^c**a**^			
Diastolic MCAv, cm·s^−1^	Eucapnia	44 ± 8^a^	−1 ± 4 (−1 ± 10)^b^	−2 ± 6 (−5 ± 13)^c^	0.25	<0.001	0.016
	5%	20 ± 9 (44 ± 21)^a^	20 ± 10 (48 ± 25)^b^	27 ± 13 (68 ± 41)^c**a**^			
Systolic BP, mm Hg	Eucapnia	129 ± 14^a^	6 ± 8 (5 ± 7)^b**a**^	12 ± 7 (10 ± 6)^c**ab**^	<0.001	0.356	0.011
	5%	8 ± 9 (6 ± 7)^a^	13 ± 7 (10 ± 6)^b^	16 ± 12 (14 ± 7)^c^^**a**^			
Diastolic BP, mm Hg	Eucapnia	61 ± 8^a^	6 ± 4 (10 ± 8)^b**a**^	8 ± 3 (14 ± 7)^c**ab**^	<0.001	0.049	<0.001
	5%	3 ± 6 (5 ± 8)^a^	9 ± 5 (15 ± 7)^b**a**^	13 ± 6 (22 ± 11)^c**ab**^			
HR, beats·min^−1^	Eucapnia	59 ± 9^a^	−3 ± 3 (−4 ± 5)^b^	−1 ± 4 (0 ± 6)^c^	0.14	<0.001	0.047
	5%	0 ± 5 (0 ± 7)^a^	1 ± 4 (3 ± 6)^b^	5 ± 5 (10 ± 10)^c^			
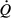 , L·min^−1^	Eucapnia	6 ± 1.4^a^	−0.2 ± 0.6 (−2 ± 11)^b^	0.0 ± 0.5 (0 ± 8)^c^	0.14	0.071	0.92
	5%	0.5 ± 0.8 (9 ± 14)^a^	−0.1 ± 0.8 (−1 ± 14)^b^	0.5 ± 1.8 (1 ± 14)^c^			
TPR mm Hg·L·min^−1^	Eucapnia	14 ± 4^a^	1.6 ± 1.7 (13 ± 11)^b**a**^	1.7 ± 1.4 (12 ± 10)^c**a**^	0.018	0.67	<0.001
	5%	−0.3 ± 2.3 (−1 ± 14)^a^	2.4 ± 3.7 (17 ± 22)^b^	2.6 ± 2.4 (17 ± 13)^c**a**^			
P_ET_CO_2_, mm Hg	Eucapnia	40 ± 3^a^	1 ± 2 (2 ± 5)^b^	−1 ± 1 (−1 ± 4)^c**b**^	<0.001	<0.001	<0.001
	5%	8 ± 3 (22 ± 10)^a^	8 ± 2 (20 ± 6)^b^	8 ± 2 (21 ± 5)^c^			

Values are absolute mean difference from baseline ±SD and percentage change from baseline values (±SD) are denoted in parentheses. The letters a, b and c represent the lower body positive pressure levels baseline (no pressure), 20 and 40 mm Hg, respectively. Multiple letters represent differences between these pressure stages within each CO_2_ intervention (*P *<**0.03). MCAv, middle cerebral artery velocity; BP, blood pressure; HR, heart rate; 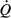, cardiac output; TPR, total peripheral resistance; P_ET_CO_2_, Partial pressure of end‐tidal carbon dioxide are shown for Pressure (P) and carbon dioxide.

Previously, we have demonstrated a divergent response for MAP and MCAv during incremental LBPP, with MCAv_mean_ decreasing at 40 mm Hg LBPP relative to 20 mm Hg despite further elevations in MAP (Perry et al. 2013). Accordingly, the eucapnic results presented here are in agreement with these previous findings that show a decreased MCAv despite the increasing MAP (Fig. 1). While we did not observe a group effect for an increase in MCAv_mean_ during mild hypertension (+20 mm Hg LBPP) in this cohort, some individual's MCAv_mean_ did increase in concert with MAP (Fig. 3). The reasons for these differences between our studies are not immediately clear, but may be possibly due to the large individual variance in cerebral autoregulatory processes (Zhang et al. 2000) and therefore differences between the recruited cohorts. Nevertheless, the decrease in MCAv_mean_ at 40 mm Hg LBPP observed here may be confounded by the small (2 mm Hg) yet significant decreases in P_ET_CO_2_ between the eucapnic 20 and 40 mm Hg LBPP stages. Given the reactivity (MCAv‐P_ET_CO_2_ sensitivity = ~2.5%) in the hypocapnic range (Ide et al. 2003; LeMarbre et al. 2003) this small decrease in P_ET_CO_2_ could account for the observed decrease in MCAv (−5%). During the eucapnic positive pressures, the trend for CVC to be lower than baseline would indicate an active regulation (vasoconstriction) against the hypertension. However, despite the greater hypertension observed with the combination of hypercapnia and positive pressures, CVC remained unchanged from hypercapnia alone (Fig. 2C). Thus, the increased MCAv responsiveness during the hypercapnic 40 mm Hg stage supports the notion that the regulatory mechanisms that would otherwise defend against the moderate hypertension observed during the eucapnic LBPPs are impaired during hypercapnia. This is consistent with previous reports that utilized pharmacologically induced increases in MAP during static autoregulatory impairment (Tiecks et al. 1995a).

Aaslid et al. (1989) demonstrated that the efficacy of dynamic autoregulation is dependent on arterial CO_2_ and therefore vascular tone. The results reported here support this notion for static autoregulation. Autoregulation is complex and thought to involve several mechanisms of action including endothelium‐dependent, myogenic and neurogenic mechanisms (Tzeng and Ainslie 2013). Likewise, although it is clear that the hypercapnia‐associated changes in pH relax cerebral vascular smooth muscle (Ainslie and Duffin 2009), the dilatory mechanisms are not entirely clear, although a role for nitric oxide has been suggested (Iadecola and Zhang 1996; Peebles et al. 2008). Regardless of the mechanisms responsible for the static regulation of CBF and how these mechanisms are impaired or modulated by hypercapnia, it is clear that hypercapnia has a profound effect on the regulatory mechanisms that are otherwise intact during eucapnia.

We have previously speculated that the moderate hypertension associated with 40 mm Hg of LBPP may induce a cerebral sympathetic response that would restrain MCAv (Perry et al. 2013). The role of the sympathetic nervous system in the regulation of the cerebral vasculature is controversial (Van Lieshout and Secher 2008). Animal models indicate a protective mechanism during acute hypertension (Bill and Linder 1976; Busija et al. 1980; Cassaglia et al. 2008). In humans the sympathetic nervous system may be tonically active and participate in beat‐to‐beat MCAv regulation (Zhang et al. 2002; Hamner et al. 2010), and may explain the asymmetric dynamic autoregulatory response between the hypo‐ and hypertensive ranges (Tzeng et al. 2010). LBPP activates intramuscular pressure receptors (Fu et al. 1998) and maintains muscle sympathetic nerve activity despite cardiopulmonary baroreceptor loading at positive pressures ≥30 mm Hg (Shi et al. 1997). Ainslie et al. (2005) reported a correlation between MSNA and cerebral resistance and it is therefore possible, yet unsubstantiated, that sympathetic modulation by LBPP may influence the cerebral vasculature.

The potential impact of sympathetic modulation on the cerebral vasculature is further confounded by the impact of hypercapnia on vessel tone. Attenuation of cerebral sympathetic nerve activity has produced a reduction in both animals (Busija and Heistad 1984) and humans (Jordan et al. 2000), no change (Peebles et al. 2012) or an increase in the slope of the P_ET_CO_2_/CBF relationship during hypercapnia. Whilst a role of the sympathetic nervous system cannot be excluded in the current conditions, the exact nature of this response remains unclear. If the increase in cerebral sympathetic activity was to persist during LBPP, this may provide a protective mechanism restraining flow during elevated CPP and decreased vessel tone in response to the LBPP and hypercania, respectively. Further research replicating the current conditions during cerebral adrenergic blockade may help elucidate the underlying mechanism.

### Technical considerations

In this experiment, transcranial Doppler ultrasound was used as a surrogate for CBF. This holds true as long as the insonated artery does not change diameter, which has been shown under various stimuli (Valdueza et al. 1997). Further, during direct observation modest increases in MAP (30 ± 16 mm Hg) resulted in small (<4%) increases in MCA diameter (Giller et al. 1993). Similarly, in this study ICA diameter increased <5% across all conditions. This is in agreement with previous data demonstrating that hypercapnia driven increases in ICA flow are mediated by changes in velocity rather than arterial diameter (Sato et al. 2012; Willie et al. 2012). Further, if there is a discrepancy between absolute flow and blood flow velocity transcranial Doppler will tend to underestimate this change (Willie et al. 2012; Liu et al. 2013). Thus, it is possible that the absolute flow responses may indeed be greater than the relative velocities reported here. Given the modest changes in MAP observed in this study, and the minimal change in diameter of the upstream vessels, it is assumed MCA diameter remained relatively unchanged by the intramural pressure. Moreover, we demonstrate this response only in the MCA territory and given the regional differences in CO_2_ regulation of blood flow (Sato et al. 2012) these results may not be heterogeneous for the entire cerebral circulation.

### Methodological considerations

As this experiment utilized LBPP to induce moderate hypertension nonpharmacologically, the number of step increases in MAP was limited. Therefore, only a two‐stepped increase in MAP was achieved and is in somewhat contrast to the multiple 10 mm Hg step increases induced pharmacologically by Lucas et al. (2010) and smaller than the step increase by Liu et al. (2013). Therefore, it is uncertain if larger increases in MAP in a background of hypercapnia will induce further increases in MCAv in a linear fashion. However, this may be limited by the ability to induce large and sustained nonpharmacological increases in MAP. In some participants MCAv nearly doubled during the hypercapnic 40 mm Hg stage, and given that the brain appears pressure passive during hypercapnia, inducing large increases in MAP during autoregulatory impairment raises serious ethical concerns.

## Conclusion

Hypercapnia has been previously demonstrated to impair dynamic cerebral autoregulation. The findings of this study support this notion for static autoregulation during nonpharmacological increases in MAP induced by LBPP. It is apparent that when MAP is elevated, over and above those induced by the chemoreceptor response alone, further increases in MCAv ensue. Despite the elevated CPP, CVC remained unchanged and showed a differential response to eucapnic LBPP where the moderate hypertension was restrained by an active regulation of the cerebral vasculature. Thus, hypercapnia impairs static cerebral autoregulation when MAP is consistently elevated by LBPP.

## Acknowledgments

The authors would like to thank the participants for taking part.

## Conflicts of Interest

None.
